# Viral-Mediated mRNA Degradation for Pathogenesis

**DOI:** 10.3390/biomedicines6040111

**Published:** 2018-11-29

**Authors:** Shujuan Du, Xiaoqing Liu, Qiliang Cai

**Affiliations:** MOE& MOH Key Laboratory of Medical Molecular Virology, School of Basic Medicine, Shanghai Medical College, Fudan University, Shanghai 200032, China; 17111010060@fudan.edu.cn (S.D.); 17211010045@fudan.edu.cn (X.L.)

**Keywords:** mRNA degradation, viral pathogenesis

## Abstract

Cellular RNA decay machinery plays a vital role in regulating gene expression by altering the stability of mRNAs in response to external stresses, including viral infection. In the primary infection, viruses often conquer the host cell’s antiviral immune response by controlling the inherently cellular mRNA degradation machinery to facilitate viral gene expression and establish a successful infection. This review summarizes the current knowledge about the diverse strategies of viral-mediated regulatory RNA shutoff for pathogenesis, and particularly sheds a light on the mechanisms that viruses evolve to elude immune surveillance during infection.

## 1. Introduction

Many human diseases, including cancer, are related to the imbalance of gene expression. mRNA degradation plays a key role in the regulation of gene expression by counterbalancing the effects of transcription. The cellular RNA decay machinery, including the mRNA deadenylation, decapping, and mRNA quality control (QC) pathways, controls the fate of RNA transcripts. Cellular RNA decay machinery plays a vital role in regulating gene expression by altering the stability of mRNAs in response to external stresses, including viral infection. In the primary infection, viruses completely rely on the host cell translation machinery, and use a variety of mechanisms (including inhibiting cap-dependent translation, transcription, and promoting host mRNA degradation) to dampen host gene expressions that are essential for viral replication. To date, it has been demonstrated that viruses have evolved several strategies to utilize cellular mRNA degradation for pathogenesis including immune evasion during viral infections.

## 2. Cellular mRNA Degradation Pathways

**Figure 1:**
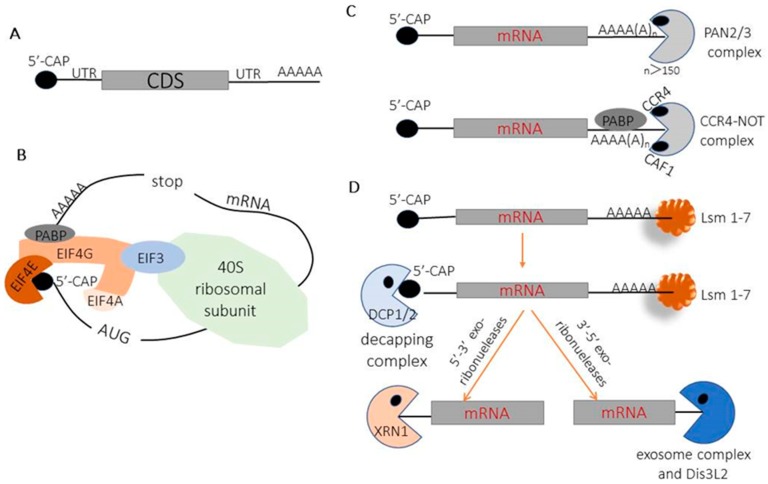
Schematics of cellular mRNA decay pathways. (**A**) Structure diagram of eukaryotic mRNAs; (**B**) conventional cap-dependent translation initiation of eukaryotic mRNA; (**C**) the degradation pathway of removal of the 3′ poly(A) tail of mature mRNA; (**D**) the decapping and degradation pathways of the mRNA body.

mRNA decay plays a vital role in transcriptional regulation. Eukaryotic mRNAs typically possess a cap at the 5′ end, 5′-UTR, CDS, and 3′-UTR, and a poly(A) tail at the 3′ end (Figure 1A). Conventional cap-dependent translation of most cellular mRNA commences with the recognition of the 7-methylguanosine cap by the eukaryotic initiation factor (eIF) 4E and the poly(A)-binding protein C1 (PABPC1) at the 3′ poly(A) tail [1]. The N terminus of eIF4G binds to poly(A)-binding protein (PABP), and the central region recruits mRNA through eIF3 binding to the 40S ribosome subunit [2,3]. The interaction between eIF4G and PABP contributes to the stabilization of mRNA by forming the closed-loop mRNA. Circularization of mRNA during translation protects mRNA from cellular decay enzymes. Thus, the 5′ 7-methylguanosine cap and the 3′ poly(A) tail serve as a ‘guardian’ by protecting mRNA from the action of exoribonucleases, and also serve to recruit translation initiation machinery (Figure 1B).

Shortening and removal of the 3′ poly(A) tail of mature mRNA is the first and rate-limiting step in mRNA degradation, which is mediated by poly(A)-specific 3′ exonucleases (deadenylases). The majority of the chemokine receptor 4 (CCR4)-NOT and PAN2-PAN3 complexes play a major role in cytoplasmic deadenylase activity [4]. Both the PAN2-PAN3 and CCR4-NOT complexes can interact with PABPC1 and be recruited to cytoplasmic mRNA for promoting mRNA deadenylation. The PAN2/3 complex removes long poly(A) tails of above 150 nt to initiate deadenylation [5]. The CCR4-NOT complex is a generic deadenylase, and its two catalytic subunits have distinct activities regarding PABPC: CAF1 trims PABPC-free A tails, while CCR4 removes PABPC-bound A tails. PABPC coordinates mRNA deadenylation and decay in a timely order by promoting deadenylation and blocking precocious decay [5] (Figure 1C). Following deadenylation, the removal of the 5′ 7-methylguanosine cap by the decapping complex DCP1/2 and its activators is triggered by the binding of the Lsm1–7 complex to the oligoadenylated 3′ end [6,7,8,9,10] (Figure 1D). After decapping, the mRNA body is degraded by the 5′ monophosphate-dependent 5′-3′ exoribonuclease 1 (Xrn1) and the 3′-5′ exoribonucleases (the exosome complex and Dis3L2) [11] (Figure 1D).

In addition to exonucleolytic decay, an mRNA can be internally cleaved in an endonucleolytic decay manner, which is a result of the mRNA QC pathway, including nonsense-mediated decay (NMD). NMD is an efficient mRNA surveillance process that selectively eliminates aberrant transcripts which harboring premature termination codons (PTCs). NMD-targeted transcripts are thought to be primarily degraded via removal of the 5′ 7-methyl guanosine cap by the decapping enzymes DCP1/2 and the subsequent 5′-3′ exonuclease activity of Xrn1 [12]. To date, it has been demonstrated that the NMD pathway is repressed by a variety of stress conditions, such as hypoxia, nutrient deprivation, or viral infection [13,14].

It is thought that RNA degradation occurs within cytoplasmic RNA granules known as processing bodies (P bodies, PBs). P bodies have been shown to contain several mRNA decay enzymes, including the DCP1/2 complex, XRN1, the Lsm1–7 complex, PAN2-PAN3, the CCR4-NOT complex, Rck/p54 (DDX6), and NUDT16, along with the P-body component GW182. Strikingly, exosome and the SKI complex proteins, involved in the 3′-5′ decay pathway, have not been observed in P bodies. P bodies are dynamic foci where protein and mRNA components are readily exchanged between the stress granules and the cytosol.

Stress granules (SGs) are dynamic and non-membrane-bound cytoplasmic compartments that arrest cytoplasmic mRNA, protein translation factors, and RNA binding proteins. SGs play a main role in regulation of mRNA translation. SGs contain stalled pre-initiation complexes (PICs), T-cell-restricted intracellular antigen 1 (TIA-1), and the two aggregation-prone RasGAP SH3-domain binding protein 1 (G3BP) and RNA-binding proteins (RBPs). It is known that cycloheximide (CHX) can bind mRNAs on polysomes and inhibit SG formation [15]. SGs assemble or disassemble rapidly, which depends on whether stress is appeared or alleviated.

## 3. Viruses Indirectly Destabilize Cellular mRNAs via Different Targets

It has been demonstrated that many cellular stresses exist including viral infection could cause different deregulation of cellular mRNA. Virus infection usually imposes stress on host cells, and hijacks the host translation machinery to ensure virus translation and production [16]. In order to establish a successful viral infection, different viruses have evolved mechanisms to disrupt the cellular decay machinery by inactivating the enzymes and co-factors, which are involved in both the constitutive and viral-induced mRNA surveillance and degradation pathways.

**Figure 2:**
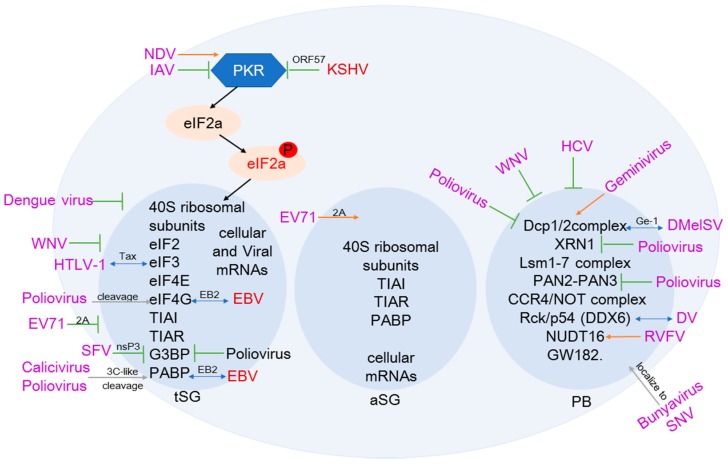
Schematics of viral-mediated regulation of mRNA degradation in stress granules and P bodies. Arrows indicate induction, T bars indicate repression.

SGs and PBs, as important structures for regulating cellular mRNA stability and translatability, have also been demonstrated to be inhibited by many viruses for viral survival (Figure 2). For examples, flaviviruses, such as West Nile virus (WNV) and dengue virus (DV), can inhibit SG formation through inducing relocalization and interaction of the cellular SG components. Although WNV infection results in a reduction in the number of P bodies, the mechanism of interference with P body assembly remains unclear [13]. The genomic RNA of dengue virus could interact with the P body component Rck/p54 (DDX6), and is presumed to be important for viral replication. Similarly, infection with hepatitis C virus (HCV) leads to a progressive reduction in the number of P body foci and relocalization of P body components to viral replication centers [17]. Picornavirus infection can also interfere with the formation of SGs. EV71-encoded 2A protease can block tSG (typical) formation but induce aSG (atypical) formation to facilitate viral translation. These aSGs are induced by cleavage of eIF4GI and are different from tSGs as they are devoid of G3BP and a series of eIFs. The aSGs are independent of eIF2α and PKR phosphorylation, and although they cannot be dissolved by CHX, it can specifically sequester cellular mRNAs instead of viral mRNAs [15]. Newcastle disease virus induces formation of bona fide in SGs to facilitate viral replication through manipulating host protein translation by activating the protein kinase R (PKR)/eIF2a pathway [18]. In addition, influenza A virus (IAV) can deploy viral nonstructural protein 1 to inhibit activation of PKR kinase and eIF2α phosphorylation, in turn blocking SG formation [19]. Semliki Forest virus (SFV) nsP3 combines its viral replication complex with host G3BP, resulting in suppression of SG formation on viral RNAs and efficient viral mRNA translation [20]. Poliovirus could prevent the assembly of SGs through the 3C-proteinase-mediated cleavage of G3BP.

In contrast to SGs, the studies on PBs in viral infection have been less comprehensive (Figure 2). Poliovirus infection leads to the loss of P bodies and the cleavage or degradation of several key proteins that are critical for cellular RNA decay, such as the P-body proteins DCP1a and PAN3. Degradation of these proteins can protect poliovirus from Xrn1-mediated antiviral response [21]. During poliovirus infection, cleavages of both eIF4GII and PABP have been proposed to contribute to host translation shutoff [22,23].

The mosquito-transmitted bunyavirus Rift Valley fever virus (RVFV) encodes nucleocapsid protein (N) to associate with P bodies for gaining access to host ribosomes by “cap-snatching” the 5′ ends of host mRNAs, while another bunyavirus, Sin Nombre virus, directly localizes to P bodies in human cells [24]. RVFV infection triggers the RNA decapping enzyme NUDT16 and selectively degrades 5′-TOP mRNAs. The increased RNA decay results in the loss of visible P bodies and stress granules. Because RVFV cap-snatches in RNA granules, the increased level of 5′-TOP mRNAs in this compartment leads to snatching of these targets, which are translationally suppressed during infection. Therefore, RVFV-induced translational arrest via decay of 5′-TOP mRNAs restricts viral infection [25].

Respiratory syncytialvirus (RSV) infection affects the expression patterns of cellular proteins by regulating mRNA translation and degradation. Following RSV infection, DCP1a is phosphorylated rapidly and this phosphorylation may modulate the expression of host chemokines in response to RSV infection [26]. DCP1 interacts with Ge-1 of Drosophila melanogastersigma virus (DMelSV) and commits mRNA for degradation by removing the 5′ cap. This RNA degradation pathway could help host cell against DMelSV infection [27]. In contrast, calicivirus 3C-like proteinase inhibits cellular translation by cleavage of PABP [23].

For herpesviruses, as shown in the Table 1, Kaposi’s sarcoma-associated herpesvirus (KSHV) inhibits SG formation by encoding a viral ORF57 protein to block PKR activation, and thus enhances virion production during lytic replication [28]. Distinct from KSHV, Epstein–Barr virus (EBV) protein EB2 stimulates translation initiation of mRNAs through directly interacting with both PABP and eIF4G [29]. Human cytomegalovirus (HCMV) encodes a noncoding RNA named as miRDE to induce the host miRNA turnover via sequence-specific noncanonical miRNA-mRNA interactions, and accelerates virus production [30]. In contrast, the human T- lymphotropic virus type 1 (HTLV-1) encodes the Tax protein to interact with both the core NMD effector UPF1 and eIF3E (a subunit of the translation initiation factor) to impair the accumulation of phosphorylated UPF1-Tax complexes in P bodies. Disruption of UPF1-INT6 association and prevention of UPF1 recycling can lead to inhibition of the NMD pathway in HTLV-1-infected cells [31].

In addition, many viruses have also evolved to counteract the interferon-induced RNase L pathway. The interferon-induced RNase L pathway is an innate immunity pathway and often is triggered by dsRNAs upon viral infection, which leads to apoptosis of the infected cells [32]. The 2′, 5′-oligoadenylate synthetase enzyme (OAS) is a upstream protein that generates the RNase L activator, 2′,5′-oligoadenylate (2-5A) from ATP. In order to overcome the degradation by cellular RNase L, many viruses encode viral dsRNA-binding proteins to inhibit the activation of OAS, such as vaccinia virus protein E3L, influenza virus NS1, HCMV proteins TRS1 and IRS1, and HSV1-encoded Us11 [32,33,34].

## 4. The Viral Proteins Directly Commandeer Cellular mRNA Turnover Pathways to Destroy Host mRNAs

Viruses have evolved different abilities to exploit the cellular mRNA decay machinery to modulate host gene expression. The description below will address the subversion mechanisms about how viruses promote host mRNA turnover (Table 2).

### 4.1. Viral Endonucleases Mediate RNA Degradation

Host shutoff is a process that virus inhibits innate immune responses and simultaneously provides preferential access for viral mRNAs to the cellular translation machinery. It has been shown that host shutoff is usually caused by viral endonucleases. For examples, influenza A virus PA-X, SARS coronavirus nsp1, or HSV-1 virion host shutoff (vhs) protein can efficiently induce endonucleolytic cleavage of the host RNA, following by RNA degradation induced by host enzymes [43].

In influenza A virus (IAV), the viral protein PA-X is an mRNA endonuclease that can restrict host gene expression through cap snatching in the nucleus [41,44]. It is generated by a ribosome frameshifting event during translation of the PA subunit of the viral RNA-dependent RNA polymerase (RdRp) [41,44,45]. PA-X can selectively target host RNA polymerase II (Pol II)-transcribed mRNAs or RNAs within the nucleus. Once activation of PA-X-mediated endonucleolytic cleavage, the complete degradation of host mRNAs is induced, which relies on the host 5′->3′-exonuclease Xrn1. Therefore, the distinct biogenesis mechanism through which IAV PA-X hijacks cellular RNA biogenesis processes for direct degradation of host RNAs provides a convenient way to discriminate host and viral products [45]. In contrast, nsp1 encoded by SARS corona virus is a potent inhibitor of host gene expression, which not only inactivates viral translation functions, but also induces host mRNA degradation by means of binding to 40S ribosomes [42].

In human herpesviruses, it is known that α-herpesvirus including HSV-1 contain a conserved virally UL41 gene that encodes a ribonuclease called the vhs. The vhs protein is directed to mRNAs through interaction with the cellular cap-binding complex eIF4F [35]. vhs possesses a potent mRNA-specific endonuclease activity that promotes endonucleolytic cleavage of viral and host mRNAs, instead of the host rRNA and tRNA [46,47]. Therefore, vhs triggers the accelerated decay of cellular mRNAs, and also destabilizes viral mRNAs. Although viral and host mRNAs are rapidly degraded, viral mRNA is transcribed at a higher rate than cellular mRNA, thus, viral mRNAs and viral proteins are not dramatically impaired by vhs protein. Similarly, PABP is partially relocated to the nucleus during HSV-1 infection, without impairing virus replication [48], the redistribution of PABP may be involved in post-transcriptional regulation of mRNA processing and/or nuclear export [49]. In contrast, although the pUL89 protein encoded by HCMV has been demonstrated as a large terminase subunit with endonucleolytic activity for genome cleavage during genome packaging and particle assembly, whether pUL89 is involved in host mRNA turnover remains obscure [30].

### 4.2. Viral Exonucleases Mediate RNA Degradation

To induce global degradation of cellular mRNAs, it has been demonstrated that gamma-herpesviruses also encode a conserved viral alkaline exonuclease, such as SOX in KSHV, muSOX in murine gammaherpesvirus 68 (MHV68), and BGLF5 in EBV, to induce host shutoff. The KSHV protein SOX is encoded by ORF37 and is able to strongly cause a widespread shutoff of cellular gene expression through enhancing global mRNA turnover [50]. The SOX-induced mRNA turnover is firstly initiated by SOX, and then degraded by exonuclease Xrn1 [51]. The SOX bound to the KSHV pre-miRNA stem loop fragment K2-31 and SOX-mediated turnover are independent of recognition of a particular consensus sequence [36]. Moreover, SOX stimulates host mRNA destruction via a unique mechanism involving polyadenylation [37]. PABPC facilitates mRNA deadenylation while preventing precocious uridylation and decay. Under steady-state conditions, PABP associates with both eIF4G and the poly(A) tail of mRNAs in the cytoplasm, thereby inducing the circularization of mRNAs. This facilitates translation initiation and hampers mRNA degradation [52]. Expression of KSHV SOX and subsequent mRNA degradation lead to the nuclear accumulation of PABP [37]. The nuclear retention of RNA transcripts could be due to a nuclear mRNA export block.

Because EBV-encoded DNase BGLF5 displays 40% homology to KSHV SOX at the amino acid level, EBV BGLF5 can also induce a robust and generalized host shutoff in productively infected cells [53]. BGLF5 can degrade mRNAs of both cellular and viral origin, irrespective of polyadenylation [54]. Similarly, EBV BGLF5 could also induce the nuclear relocation of PABP. Within the nucleus compartment, PABPC causes hyperadenylation and retention of nuclear mRNA molecules, thereby augmenting the shutoff phenotype initiated by BGLF5 [37]. In contrast, MHV68-encoded muSOX also induces both cellular and viral mRNA degradation upon productive infection [38]. However, the association between muSOX and PABP remains unknown.

### 4.3. Viral Decapping Enzymes Mediates RNA Degradation

In order to inhibit the host antiviral responses, it has been shown that some poxviruses, such as vaccinia virus (VACV), can encode a Nudix hydrolase motif-containing mRNA decapping enzymes D9 and D10, to remove protective caps from mRNA 5-termini by preventing dsRNA accumulation and shutdown of host protein synthesis [55]. The D9- or D10-deficient VACV are effective oncolytic viruses, and their VACV mutants unable to execute a fundamental step in virus-induced mRNA decay [40]. Similarly, African Swine Fever Virus (ASFV) also contains a gene (D250R in strain Ba71V and g5R in strain Malawi) to encode a decapping protein (ASFV-DP) that has a Nudix hydrolase motif and decapping activity in vitro [39,56]. Interestingly, g5R binds to the RNA body rather than the cap, whereas VACV D10 binds both the methylated cap and the RNA body. ASFV-DP was expressed from early times and accumulated throughout the infection with a subcellular localization typically in the endoplasmic reticulum, and co-localizing with ribosomal protein L23a in the cap structure. Moreover, the N-terminal region of the ASFV-DP protein has been shown to interact with poly(A) RNA of both viral and cellular RNAs in the infected cells, which results in decreased transcripts levels [39].

## 5. Cellular mRNA Degradation Contributes to Immune Evasion

To establish a successful infection, it is well known that RNA viruses must contend with the antiviral response. Many viruses have evolved variety of strategies. These include directly preventing the synthesis of antiviral associated proteins or forming special structures with cellular RNA binding proteins or enzymes that can impede the function of RNA degradation, to disrupt and inhibit the decay machinery or redirect decay machinery. How viruses take advantage of host shutoff pathways to evade antiviral immunity has also been broadly investigated. For examples, EBV BGLF5 can reduce expression of HLA class I and II molecules, thereby hampering antigen presentation to T cells [54,57]. Meanwhile, BGLF5-mediated shutoff can reduce expression of the innate EBV-sensing Toll-like receptor-2 and the lipid antigen-presenting cluster of differentiation 1 (CD1d) [58]. In contrast, HSV-1 vhs-mediated shutoff of host protein synthesis occurs mainly in the immediate-early and early phases of lytic infection. In this phase, vhs reduces synthesis of proteins involved in the innate and adaptive immune responses, including dampen the type I interferon (IFN) system. This helps HSV-1 resist eradication by the immune system. Recent studies showed that vhs also targets the Cyclic GMP-AMP (cGAMP) synthase cGAS/STING-mediated cellular DNA-sensing pathway by selectively degrading cGAS mRNA [59]. This suggests that HSV-1 not only can evade immune evasion but also block the cytosolic DNA sensing and signaling.

In addition, viruses have also developed different ways to repress or avoid deadenylation which is often the key step in mRNA decay. Many RNA viruses have evolved 3′ terminal stem loop structures to maintain the stability of the transcript and the translational ability [60]. For examples, SCoV mRNAs, which have a 5′ cap structure and 3′ poly A tail like those of typical host mRNAs, are not susceptible tonsp1-mediated RNA cleavage, while the presence of the 5′-end leader sequence could protect the SCoV mRNAs fromnsp1-induced endonucleolytic RNA cleavage. In contrast, some positive-sense single-stranded RNA viruses, such as Sindbis virus (SINV) and Venezuelan equine encephalitis virus (VEEV) whose genomic RNAs are both capped and polyadenylated and resembling cellular mRNAs, have evolved sequences that can stall deadenylation [61]. After deadenylation, the process of RNA degradation is completed through the 5′-3′ or 3′-5′ decay pathway. Different RNA viruses have evolved different or similar strategies to avoid the mRNA decay pathways. For instances, Poliovirus contains an RNA element to interact with and inactivate the RNase L endonuclease [62]. A pseudoknot structure stalling the XRN1 enzyme is presented at the 5′ border of the 3′ UTR of insect-borne flaviviruses [63]. Rous sarcoma virus also contains an RNA element that insulates unspliced viral mRNAs from the nonsense-mediated decay pathway [64]. Moreover, it has been demonstrated that the rate of decay of specific mRNAs can be controlled by using RNA structures. For example, a thermostable RNA secondary structure at the 5′ or 3′ end of RNA could slow down the degradation machinery. Further investigation revealed that some RNA forms could fold subtle three-dimensional conformations to confound or evade the decay machinery [65]. Interestingly, in order to protect themselves from host immune detecting, some negative-sense RNA viruses may steal stability factors from host mRNAs to incorporate into their own transcripts. For example, rabies virus steals host poly(C) binding protein 2 (PCBP2) to regulate expression of its glycoprotein to avoid host immune detection as it replicates and migrates to the central nervous system during infection [66]. Taken together, this evidence indicates that viruses have evolved a number of strategies to avoid or inactivate the cellular mRNA decay pathways and direct an optimum cellular environment for their own replication.

## 6. Conclusions

In summary, mRNA decay is important for regulation of cellular gene expression. In order to facilitate viral replication, the virus exploits multiple strategies to corrupt host shutoff through RNA degradation or altering cellular RNA metabolism. mRNA released from disassembled polysomes is sorted and remodeled at SGs; the accumulation of mRNA at SGs may be a consequence of both stress-induced translational arrest and virus-induced host shutoff, from which selected transcripts are delivered to PBs for degradation. Blocking protein synthesis could benefit virus infection. In the absence of host protein synthesis, cellular ribosomes will be committed to the synthesis of viral proteins. Taking advantage of this point, the virus can progress efficiently between the different phases of productive infection by controlling the transition between immediate early, early, and late protein synthesis. Given the essential role of mRNA decay in antiviral defense, host shutoff contributes to immune evasion by preventing the synthesis of proteins involved in antiviral immunity. To fight with the host cell’s antiviral immune response, viruses have evolved multiple strategies including encoding the viral counterpart of endonucleases, exonucleases, and decapping enzymes that can directly commandeer cellular mRNA turnover pathways to destroy host mRNAs, which will facilitate viral gene expression and eventually establish a successful infection.

## Figures and Tables

**Table 1:** Molecular mechanisms of viruses indirectly destabilize cellular mRNAs. KSHV: Kaposi’s sarcoma-associated herpesvirus; EBV: Epstein–Barr virus; HCMV: Human cytomegalovirus; RSV: respiratory syncytialvirus; DMelSV: Drosophila melanogastersigma virus; IAV: influenza A virus; SFV: Semliki Forest virus. HTLV-1: human T- lymphotropic virus type 1; RVFV: Rift Valley fever virus.

Viruses	Viral Antigen	Host Shutoff Factor(s)	Targeting Mechanisms	Refenrence
DNA viruses				
KSHV	viral ORF57	PKR	Inhibits stress granule formation	[28]
EBV	EB2	PABP and eIF4G	EB2 directly interacts with both PABP and eIF4G	[29]
HCMV	miRDE in UL144-145 region	host miRNA	miRNA–mRNA interactions	[30]
RNA viruses				
RVFV	viral nucleocapsid protein (N), viral polymerase (L)	P bodies	N “cap-snatching” the 5′ ends of host mRNAs, and L cleaved 10–18nt downstream of the 5′ cap. This capped oligomer is used for viral transcription. RVFV N associates with P bodies	[24,25]
DMelSV	Ge-1	DCP1	DCP1 interacts with Ge-1	[27]
RSV		DCP1 phosphorylation	Inhibits IL-8 expression	[26]
IAV	nsP1	PKR	Blocking SG formation	[19]
SFV	nsP3	Ras-GAP	Suppression of SG formation	[20]
Poliovirus	3C-proteinase	G3BP, DCP1, PAN3	Prevent the assembly of SGs and disrupt PBs	[21]
HTLV-1	Tax	UPF1, INT6/EIF3E	Effect the accumulation of phosphorylated UPF1-Tax complexes in P bodies	[31]

**Table 2:** Molecular mechanisms of viral enzymes directly destroy gene expression.

Viruses	Viral Protein	Viral Gene	Targeting Mechanisms	Reference
DNA viruses				
HSV1	Virion host shutoff protein (vhs)	*UL41*	eIF4H and eIF4AI/II	[35]
KSHV	SOX	*ORF37*	KSHV pre-miRNA stem loop fragment K2-31	[36]
EBV	BGLF5	*BGLF5*	Nuclear relocalization of PABPC1	[37]
HCMV	pUL89	*UL89*	the endonucleolytic activity for virus genome cleavage.	[30]
MHV68	muSOX	*ORF37*	Unknown	[38]
RNA viruses				
ASFV	ASFV-DP	*Ba71V D250R/Malawi g5R*	poly(A) RNA RPL23a	[39]
VACV	decapping enzymes	*D9*, *D10*	Cap-binding	[40]
IAV	PA-X	*PA*	selectively targets host RNA polymerase II (Pol II) transcribed mRNAs and non-coding RNAs	[41]
SARS	nsP1	*ORF1AB*	binding to 40S ribosomes, inactivate the translation functions	[42]
